# The Role of Thalidomide and Its Analogs in the Treatment of Hereditary Hemorrhagic Telangiectasia: A Systematic Review

**DOI:** 10.3390/jcm13185404

**Published:** 2024-09-12

**Authors:** Mehmet Can Ugur, Mehmet Baysal, Elif Gulsum Umit

**Affiliations:** 1 Division of Hematology, Department of Internal Medicine, Çiğli Training and Research Hospital, İzmir Bakırçay University, 35665 İzmir, Turkey; med.can@hotmail.com; 2 Division of Hematology, Ali Osman Sönmez Oncology Hospital, 16040 Bursa, Turkey; 3 Division of Hematology, Department of Internal Medicine, Faculty of Medicine, Trakya University, 22030 Edirne, Turkey; egugur@yahoo.com

**Keywords:** hereditary hemorrhagic telangiectasia (HHT), thalidomide, pomalidomide, lenalidomide, Osler–Weber–Rendu

## Abstract

**Background:** Hereditary hemorrhagic telangiectasia (HHT) is a disease characterized by arteriovenous malformations and telangiectases, in which the endothelium and immune system play a role in the pathophysiology. Therefore, treatments with antiangiogenic properties which are also regarded as immunomodulators were demonstrated to play an important role in treatment. This systematic review aimed to gather the accumulated information of the use of thalidomide and its analogs in the treatment of HHT. **Methods:** In this systematic review, publications that were published up to March 2024 and met the inclusion criteria were compiled using the keywords ‘thalidomide’, ‘lenalidomide’, ‘pomalidomide’, ‘immunomodulatory drugs’ and ‘HHT’ in Medline and Scholars databases. **Results:** A total of 53 articles were evaluated and 15 were included in the study. Thalidomide was the predominant used agent and was observed to be used in patients with ages ranging from 37 to 77 years, with doses ranging from 50 to 200 mg daily, and the mean follow-up period was observed to be 6–60 months. Assessments regarding efficacy were based on the epistaxis severity score (ESS), hemoglobin level, and transfusion independence. While thalidomide showed significant efficacy, it also had an adverse event rate of any severity of up to 85% of patients. Use of lenalidomide to control bleeding in HHT was reported in a single case report, while the use of pomalidomide was observed to be investigated in Phase 1 and Phase 2 studies in patients aged 48 to 70 years, with doses ranging from 1 to 5 mg daily for 6–24 months. This treatment was reported to provide significant improvement in hemoglobin levels and ESS. Adverse events of any severity were observed at a frequency of 60–66%. **Conclusions**: Antiangiogenic agents such as thalidomide, lenalidomide, and pomalidomide may be effective in managing HHT. However, further studies are needed to optimize the timing, dose, and sequence.

## 1. Introduction

Hereditary hemorrhagic telangiectasia (HHT), also known as Osler–Weber–Rendu disease, is a rare autosomal dominant disease characterized by the development of arteriovenous malformations (AVMs) and telangiectases. The latter can be found in various organs, including the nose, gastrointestinal tract, and skin, and may present with epistaxis and gastrointestinal bleeding. Vascular abnormalities may also occur in the liver, lungs, or brain, and may lead to high morbidity and mortality in affected individuals [1,2]. HHT affects approximately 1 in 5000 individuals in North America, but because the diagnosis is frequently missed, the true prevalence is unknown [3]. The most important complication of HHT is anemia due to chronic bleeding and AVM. Approximately 50% of the affected patients have anemia. Additional risk factors such as epistaxis and gastrointestinal bleeding intensity, menstrual bleeding in women, and comorbidities in the elderly may trigger the development of anemia [4]. The deepening of these complications and symptoms may affect the patients’ daily lives, social relationships and professional lives, reducing their quality of life. Oral or parenteral iron replacements and erythrocyte transfusions are the most important types of hematologic support treatment in HHT. These treatments also cause a burden as well as bring risks of adverse events. Therefore, to provide a standardized definition of these treatments, a scoring system calculated by the number of transfusions and the amount of elemental iron, the Hematologic Support Score (HSS), was proposed by Al-Samkari et al. [5]. 

Quality of life is often impaired due to bleeding and bleeding-associated iron deficiency. Geisthoff et al. have reported a significant relation between the duration of epistaxis, gastrointestinal bleeding, presence of telangiectases within the liver, as well the number of detectable telangiectases with HR-QoL in patients with HHT [6]. Factors affecting the quality of life in HHT patients should be thoroughly investigated to evaluate the impact of HHT on certain aspects of patients’ quality of life and to provide patients with individualized medical and psychosocial support.

HHT is diagnosed clinically by the Curacao Criteria [7]. If the following three criteria are met, a diagnosis is made:

Spontaneous, recurrent epistaxis.

Multiple telangiectases at characteristic sites.

Visceral AVMs.

A first-degree relative with HHT according to these criteria.

A pathogenic sequence variant also defines clinical HHT and a negative gene test does not exclude it. 

The pathogenesis of HHT is mainly attributed to mutations in genes such as *Endoglin (ENG)*, *Activin receptor-like kinase 1 (ACVRL1/ALK1)*, and *SMAD4*. *ENG e* and *ALK1* are cell-surface glycoproteins that function as part of the transforming growth factor beta (TGF-β) signaling complex that plays an important role in angiogenesis and vascular remodeling. *SMAD4* is a transcription factor that mediates signal transduction in the TGF-β pathway and it is related to juvenile polyposis [8,9]. Among HHT patients, over 90% have *ENG* or *ACVRL1* mutations, while approximately 2% have *SMAD4* mutations and, in rare instances, *GDF2 (BMP9)* mutations [8,9].

The genes involved in the pathogenesis of HHT are also responsible for inhibiting vascular endothelial growth factor (VEGF), a factor that contributes to angiogenesis. Mutations in these genes are demonstrated to be related to increased VEGF levels [10]. In addition to these mutations, environmental stimuli such as inflammation, hypoxia, neoangiogenesis, vascular injury, shear stress, radiation, or trauma may be related to the clinical manifestations of HHT and may be regarded as another “hit” [10,11]. Chronic iron deficiency or interruption in iron mobilization may also contribute to or be related to neovascularization [10]. These genetic and environmental stimuli may lead to complex interactions between vascular endothelial cells and the immune system, which are mainly derived from hemangioblasts [11]. Antiangiogenic agents, such as thalidomide and its analogs, have been investigated for their potential in managing HHT-related symptoms [11,12].

Thalidomide and its further generations, whose antiangiogenic effects had tragic consequences in the 1950s, attracted attention to the treatment of HHT for these very severe adverse events. The effectiveness of thalidomide, the first agent in this group, has attracted considerable attention in the treatment of HHT with the significant reduction in bleeding in patients with angiodysplasia and HHT [13]. In recent years, there has been growing interest in exploring the efficacy and safety of various immunomodulatory drugs (IMIDs) in managing HHT-related complications. These agents target key immune pathways implicated in the pathophysiology of HHT, including inflammation, angiogenesis, and endothelial dysfunction [14,15]. Despite the promising preclinical data and anecdotal reports supporting their use, the clinical evidence regarding the effectiveness of IMIDs in HHT remains limited and heterogeneous.

Given the complexity of HHT and its diverse manifestations, a comprehensive evaluation of the existing literature is essential to elucidate the role of immunomodulatory therapies in managing this condition. Therefore, we conducted a systematic review to synthesize the available evidence on using thalidomide and its analogs in HHT and evaluate their impact on disease outcomes, focusing on safety, efficacy, and potential mechanisms of action. By critically appraising the current literature, we aim to provide insights into the therapeutic landscape of HHT and identify areas for future research and clinical intervention.

## 2. Materials and Methods

**Figure 1:**
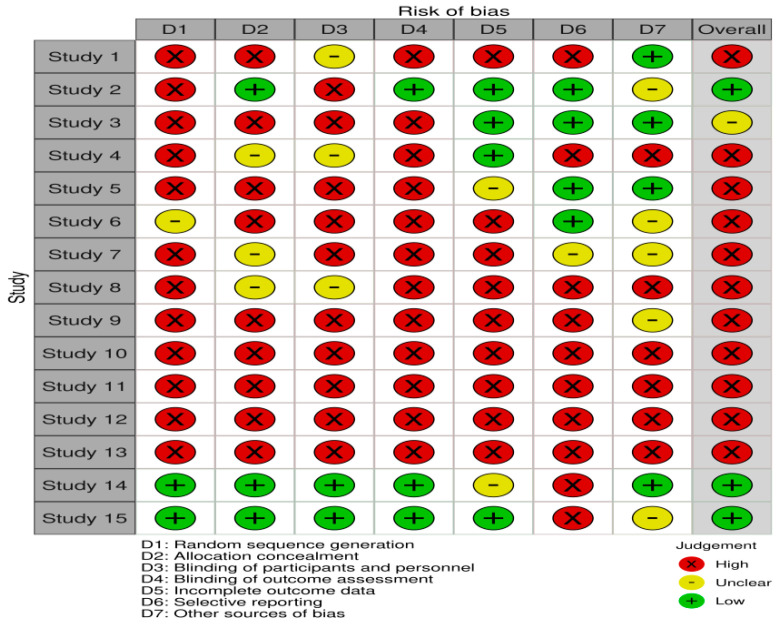
Robvis assessment tool to evaluate the risk of bias.

This systematic review adheres to the Preferred Reporting Items for Systematic Reviews and Meta-Analyses (PRISMA) guidelines [15]. A review protocol was prepared before the study began and was applied throughout the study. A comprehensive literature search of Medline, PubMed, Web of Science, Embase, Scopus, and Scholars databases was performed until March 2024, identifying all studies relevant to the question of interest. The search strategy employed a comprehensive set of keywords to identify relevant studies using IMIDs in hereditary hemorrhagic telangiectasia (HHT). Keywords included ‘thalidomide’, ‘lenalidomide’, ‘pomalidomide’, ‘immunomodulatory drugs’, and ‘HHT’. A literature search was conducted by two writers who independently examined the entire texts of eligible studies to determine if they matched the inclusion criteria. Any discrepancies were settled by discussion amongst the authors, with the involvement of the senior author if necessary for the decision-making process. The Robvis assessment tool (visualization tool for risk of bias assessments in a systematic review) was used to evaluate the risk of bias (Figure 1). This review has not been registered in any publicly accessible database

### 2.1. Inclusion and Exclusion Criteria

Studies were selected according to pre-defined eligibility criteria, including meta-analysis, randomized controlled trials, observational studies, case reports, and conference proceedings. The inclusion criteria included studies that evaluated the effectiveness and safety of immunomodulatory drugs in patients diagnosed with HHT. The included publications met the Curaçao criteria or contained diagnoses that had been confirmed by genetic testing, and they included information about patients who discontinued treatment, such as their age, drug dose, treatment duration, and ESS, as well as an evaluation of each patient’s clinical condition before and after treatment, their hemoglobin level, at least one of the transfusion requirement parameters, and the frequency of their side effects and discontinuation of treatment. Studies that did not fit these parameters were excluded.

### 2.2. Evaluation Method

In the included studies, the age range of the patients (in years), use of thalidomide, lenalidomide, and pomalidomide doses, duration (months), disease symptoms, the change in symptoms with treatment, the rate of side effects (%), and whether these side effects led to discontinuation of therapy were evaluated. Regarding the efficacy of the treatments, the epistaxis severity score (ESS), hemoglobin change, and change in transfusion need were also assessed.

## 3. Results

**Figure 2:**
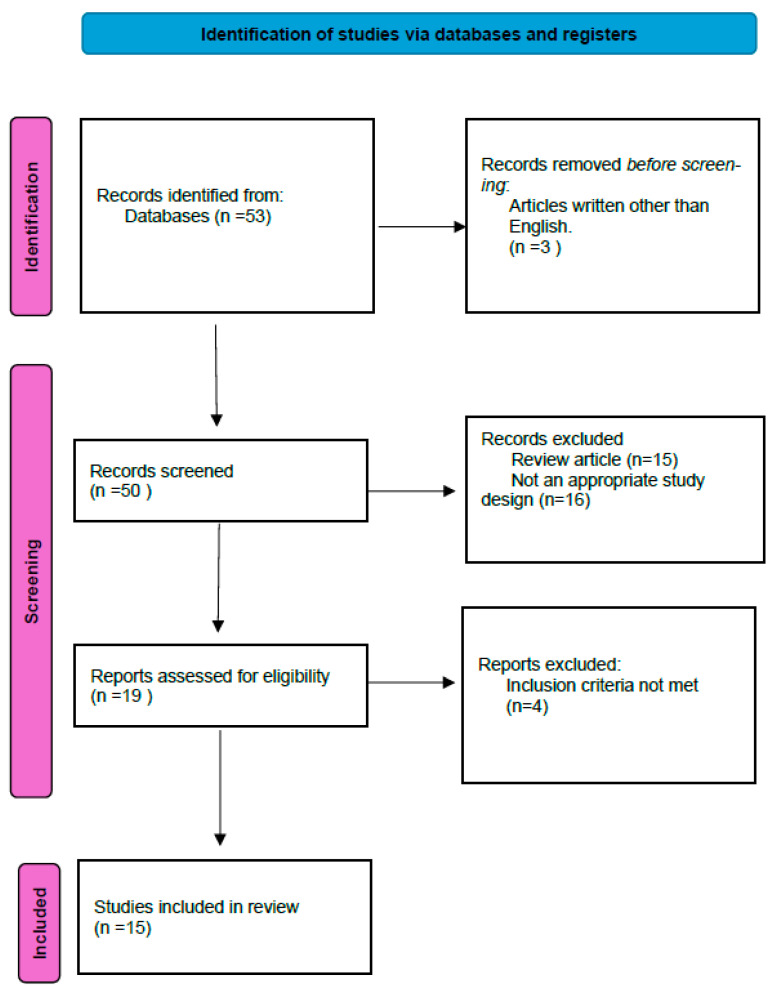
Preferred Reporting Items for Systematic Reviews and Meta-Analyses (‘PRISMA’, see Supplementary Materials File S1) flow chart showing the identification of studies.

For this systematic review, 53 publications were screened and 15 publications that met the inclusion criteria were included (Figure 2). It has been observed that thalidomide has been more widely researched as a treatment for HHT. Of the 15 studies included in this review, 12 were thalidomide studies. These studies are summarized in Table 1.

The mean age of patients involved in thalidomide studies was observed as 37–77 years. The thalidomide dose was 50–200 mg/day and the average treatment duration ranged from 6 to 60 months. The treatment response was evaluated in studies by clinical monitoring of ESS or epistaxis, hemoglobin increase, reduction in the transfusion requirement, and achievement of transfusion independence. In thalidomide studies, response to treatment was indicated by a decrease in epistaxis in four studies, a decrease in the need for transfusion in six studies, and an increase in hemoglobin in three studies. In thalidomide studies, a decrease in epistaxis was found in four studies, a decrease in the need for transfusion was found in six studies, and an increase in hemoglobin was observed in three studies, indicating a response to treatment. In the study by Fang et al. [16], the median ESS decreased from 5 to 0.9, and in Baysal et al.’s [20] study, it decreased from 7.4 to 3.1. In Baysal et al.’s study, the median hemoglobin value increased from 8.8 g/dL to 11.5 g/dL; in Lebrin et al.’s [22] study, it increased from 6.0 to 7.4 g/dL; and in Alam et al.’s [23] study, it increased from 5.8 g/dL to 13.0 g/dL. The median number of red blood cells transfused decreased from 23 to 3 in Invernizzi et al.’s [17] study, from 3.7 to 0.5 in Hosman et al.’s [21] study, and from 43 to 1 in Alam et al.’s [23] study. In all of these studies, clinical benefit was reported to be observed, while in the study of Buscarini et al., it was stated that thalidomide was not effective in patients with AVM. In pomalidomide studies, Samour et al. [28] reported a more than 1 g/dL increase in the median hemoglobin value and a 50% decrease in ESS, while Al-Samkari et al. [29] reported a significant decrease in ESS compared to placebo.

While individual case reports may indicate a lack of reported side effects, larger-scale studies have demonstrated an occurrence rate of up to 85% for various adverse events. Notably, these adverse effects have been documented as severe and potentially necessitating the cessation of treatment. A total of 10 patients (10.5%) in thalidomide studies and 26 patients (25.7%) in pomalidomide studies discontinued treatment due to adverse events. Although a wide spectrum of different adverse effects was observed in the studies, the most frequent adverse events were neuropathy and gastrointestinal problems. The most common adverse effects in the Phase 2 study of thalidomide were neurological symptoms such as paresthesia and asthenia. Asymptomatic TSH elevation was observed in five cases. Despite being one of the major concerns for adult patients, no thromboembolic events were recorded [17]. In another large sample study including 67 patients, the most common side effects were somnolence, drowsiness, peripheral neuropathy, cardiac failure, and thromboembolic events in 2.9% of patients [18].

No study has been found on the use of lenalidomide in HHT, which is superior to thalidomide in terms of efficacy and safety in different disease groups such as Multiple Myeloma. In a 69-year-old HHT case report published by Bowcock and Patrick [30], after thalidomide was discontinued due to neuropathy, lenalidomide was used at a dose of 25 mg/day with titration, starting from a dose of 10 mg/day. No gastrointestinal bleeding was observed with lenalidomide, and a decrease in iron requirement and an increase in hemoglobin level were observed. Although lenalidomide-related cytopenias were observed, there were no complications and there was no need to discontinue the drug.

A Phase 1 study on the use of pomalidomide for the treatment of HHT was presented at the ASH Congress in 2016 [28]. This study included six patients between the ages of 48 and 70 and one patient withdrew from the study before the treatment started. Patients started at a dose of 1 mg/day and increased to a maximum dose of 5 mg/day. The patients’ treatment continued for more than 6 months. During this period, treatment of two patients was discontinued due to treatment-unrelated reasons, and one patient discontinued treatment due to a Pomalidomide-related rash. An increase in hemoglobin of more than 1 g/dL, a decrease in iron requirement, and a more than 50% decrease in ESS was observed in patients with Pomalidomide.

In the PATH-HHT study [29], a Phase 2 study investigating the effectiveness of pomalidomide in the treatment of HHT, 95 patients were randomized to the pomalidomide arm and 49 patients were randomized to the placebo arm. The mean age of the patients was 58.8 years. After 24 weeks of follow-up, the ESS decreased significantly in the pomalidomide arm (*p* = 0.003). The adverse events observed in the pomalidomide arm were gastrointestinal symptoms (60%), neutropenia (45%), and rash (36%). The discontinuation rate in this arm was 25%. The publications relating to pomalidomide are summarized in Table 2.

## 4. Discussion

The common experience with thalidomide analogs in the treatment of HHT was observed as a result of treatment with thalidomide. While the exact mechanism by which thalidomide acts within the angiogenic cascade has not yet been determined, increased expression of integrin genes and decreased VEGF levels were considered as probable pathways [31]. Also, it has been shown that TGF-β3 messenger RNA and plasma VEGF protein expression decreased after thalidomide administration in the transgenic zebrafish model [32]. In another disease model of mice heterozygous for a null mutation in the *ENG* gene, thalidomide increased platelet-derived growth factor-B (PDGF-B) expression in endothelial cells and stimulated mural cell activation. Additionally, it increased the number of pericytes and their recruitment to blood vessels, enhancing the opposition between the inner endothelial and supportive pericyte layers and resulting in vessel stabilization. These results demonstrate the ability of thalidomide to induce vascular maturation [22]. It was observed that studies with relatively large sample numbers were published at older dates, while current thalidomide experiences were published as case reports. In a meta-analysis, treatment with thalidomide resulted in a significant improvement in the frequency, intensity, and duration of epistaxis [33].

Although lenalidomide has stronger biological activity and a safer side effect profile compared to thalidomide, it has not been as widely studied as thalidomide as a treatment for HHT [34]. However, the fact that lenalidomide is effective in pathologies other than HHT, which progresses with angiodysplasias such as von Willebrand Disease, shows that it has potential for the treatment of HHT [35].

The Phase 2 study of pomalidomide suggests that pomalidomide also has an important place as an immunomodulatory agent in the current HHT treatment protocol Based on the results of the pomalidomide study, in which a large number of patient groups are compared and randomized controlled phase studies are published, it is seen that immunomodulatory agents are effective at treating HHT. When these agents are used, better control of epistaxis is achieved, the need for transfusion decreases, and the average hemoglobin level increases.

In HHT patients, chronic inflammation, endothelial damage, shear stress, increased FVIII, and von Willebrand factor levels due to bleeding bring about an increase in the risk of thrombosis. The impaired filter function in patients with pulmonary arteriovenous malformations is the main risk factor for thromboembolism/stroke. In addition, iron deficiency in these patients may also be associated with the development of thrombosis. Studies showing that the frequency of deep vein thrombosis and pulmonary thromboembolism increases in people with HHT support this increased risk [36]. In addition to this thrombotic risk, there is additional thrombosis risk caused by the immune modulator drugs used in treatment. Thalidomide has also been shown to trigger intrinsic thrombin formation by thrombin generation testing [37]. In HHT patients, antithrombotic and anticoagulant treatment due to thrombosis provokes bleeding. In order to control bleeding, patients may need to be treated with local ablative treatments or antiplatelet/anticoagulant treatment may need to be discontinued. The risk of bleeding increases especially in patients over the age of 60, those requiring hospitalization due to bleeding or transfusion, those with a history of gastrointestinal bleeding, and those receiving dual antiplatelet therapy [38]. However, there is not enough information in the literature about thromboprophylaxis with IMIDs treatment in HHT patients. There were insufficient data on this subject in the publications included in our systematic review. Moreover, arteriovenous malformations and recurrent bleeding create a dilemma in terms of thromboprophylaxis.

Another dilemma associated with thalidomide and its analogs for the treatment of HHT relates to the risk of infection. In HHT, neutrophil dysfunction and a decrease in the T cell count are observed due to possible inflammatory processes, although the mechanism has not yet been clearly defined [39]. Information about managing the risk of infection with these agents, which are also immunosuppressive, is not adequately covered in the literature.

Al-Samkari also has recommended thalidomide and bevacizumab, which are both antiangiogenic drugs that reduce VEGF levels, in cases where local treatments are not sufficient. However, due to the wide side effect profile of thalidomide, long-term use of this drug is not recommended [40]. International working groups and guidelines also recommend bevacizumab for the systemic treatment of HHT [41,42]. However, the frequent hospital admissions in patients taking bevacizumab, the fact that this agent has side effects such as thrombosis, and the lack of head-to-head comparative studies with oral agents keep thalidomide and new-generation analogs on the agenda in the treatment of HHT. All these discussions reveal the need to determine the place of thalidomide and its analogs in HHT treatment.

Studies on drugs that may have potential for treating HHT in the future are ongoing. The drugs current under study are Sorafenib (a dual Raf kinase/VEGF receptor inhibitor), Pazopanib (a multi-target tyrosine kinase inhibitor with anti-VEGF receptor properties) and Tacrolimus (an activator of the ALK1-SMAD1/5/8 pathway), which are also thought to be effective for treating VEGF [43,44]. Another potential hypothesis is that acquired von Willebrand syndrome occurs due to loss of large molecular multimers of VWF due to increased shear stress and that bleeding in HHT can be reduced via von Willebrand factor replacement [45]. In the study by Yadav et al., AVM formation in brain endothelial cells was prevented by intravenous transfer of the gene of interest to mice with ENG mutation via hepatotropic adeno-associated virus (AAV). This result is promising for future human experiments [46].

In addition to medical advancements in the treatment of HHT, which is a multisystemic disease, there is also a need for progress in the organization of disease management. Specialists in multiple fields may be called to care for one or more aspects of HHT, but few are familiar with evidence-based guidelines for HHT management or see a sufficient number of patients to gain experience with the unique characteristics of the disease. Primary care physicians and specialists are often unaware of the important manifestations of HHT in multiple systems and the thresholds for their screening and appropriate management. The publication by Alkhalid et al. may also contribute to this research area [47].

The limitations of this review are that there are few publications on the use of thalidomide and its analogs in the treatment of HHT, the existing publications consist of small samples or case reports, and the majority of the publications are retrospective studies. A meta-analysis was not possible because HHT symptoms, the doses and durations of treatments given, and adverse events encountered were not evaluated homogeneously in the chosen publications.

## 5. Conclusions

Thalidomide and its analogs may be effective for managing symptoms of HHT. However, prospective, comparative, and long-term studies are required to determine the safety and efficacy of these treatments.

## Figures and Tables

**Table 1:** Studies evaluating thalidomide in hereditary hemorrhagic telangiectasia.

Study	No. of Patients	Age, y	Thalidomide Dose, mg/d	Duration of Treatment (m)	Treatment Effect Evaluation		Side Effects	
					Before	After	Incidence Rate (%)	Stopped Treatment Because of Side Effects
**Fang et al.** [16]	**7**	**49.1**	**50–100**	12.8	ESS: 5	ESS: 0.9	57	1
Invernizzi et al. [17]	31	62.6	50–150	15.9	Total erythrocytes transfusion: 23	Total erythrocytes transfusion: 3	58	0
Buscarini et al. [18]	67	66.4	100–200	13.4	Reducing Transfusion dependency, GI bleeding, and epistaxis. No effect in AVM.		55	3 fatal AE
Balduini et al. [19]	11	67	50–200	11.0	Reducing transfusion dependency and epistaxis; increasing hemoglobin level		Nonserious constipation and drowsiness	
Baysal et al. [20]	6	60.5	50–100	8	ESS: 7.4	ESS: 3.1	33	0
					Hg: 8.8 g/dL	Hg: 11.5 g/dL		
					Erythrocytes transfusion: 5/month	Erythrocytes transfusion: 0.8/month		
Hosman et al. [21]	12	69.5	50–100	7	Number of epistaxis: 40.8/week	Number of epistaxis: 5.6/week	66	7
					Total erythrocytes transfusion: 3.7	Total erythrocytes transfusion: 0.5		
Lebrin et al. [22]	7	Range: 48–75	100	* 6–60	Hg: 6.0 g/dL	Hg: 7.4 g/dL	85	3
Alam et al. [23]	1	77	100	16	Hg: 5.8 g/dL	Hg: 13.0 g/dL	0	0
					Total erythrocytes transfusion: 43	Total erythrocytes transfusion: 1		
Amanzada et al. [24]	1	59	200	29	Reducing transfusion dependency		100	1
Chen et al. [25]	1	38	50	6	Erythrocytes transfusion frequency: 3 times a week	Erythrocytes transfusion frequency: once a month	0	0
Nakamura et al. [26]	1	37	50	12	Reducing transfusion dependency		100	1
Wang et al. [27]	1	77	100	12	Reducing epistaxis		0	0

* It was written according to the wording stated in the original article. AE: adverse event, m: month, mg/d: milligram/day, y: year.

**Table 2:** Studies evaluating pomalidomide in hereditary hemorrhagic telangiectasia.

Study	No. of Patients	Age, y	Pomalidomide Dose, mg/d	Duration of Treatment (m)	Treatment Effect Evaluation	Side Effects	
						Incidence Rate (%)	Stopped Treatment Because of Side Effects (%)
**Samour M et al.** [28]	**6**	**min: 48–max: 70**	**min: 1–max: 5**	**6**	Increasing in hemoglobin of more than 1 g/dL decreasing of more than 50% in ESS	66	50
Al-Samkari H et al. [29]	95	58.8	4	24	Decreasing in ESS compared to placebo (*p* = 0.003)	60	25

## Supplementary Materials

The following supporting information can be downloaded at https://www.mdpi.com/article/10.3390/jcm13185404/s1, File S1: PRISMA 2020 Checklist.
